# ^68^Ga-DOTA-FAPI-04 PET/CT as a Promising Tool for Differentiating Ovarian Physiological Uptake: Preliminary Experience of Comparative Analysis With ^18^F-FDG

**DOI:** 10.3389/fmed.2021.748683

**Published:** 2021-10-04

**Authors:** Qixin Wang, Songsong Yang, Wenxin Tang, Lin Liu, Yue Chen

**Affiliations:** ^1^Department of Nuclear Medicine, The Affiliated Hospital of Southwest Medical University, Luzhou, China; ^2^Nuclear Medicine and Molecular Imaging Key Laboratory of Sichuan Province, Luzhou, China

**Keywords:** ^18^F-FDG, ^68^Ga-DOTA-FAPI-04, PET/CT, ovary, physiological metabolism

## Abstract

**Objectives:** This study aimed to investigate the physiological distribution characteristics of ^68^Ga-DOTA-FAPI-04 in the ovary, and assess the feasibility of early diagnosis of primary ovarian disease with ^68^Ga-DOTA-FAPI-04 PET/CT.

**Methods:** We retrospectively analyzed the data of patients who received ^18^F-FDG and ^68^Ga-DOTA-FAPI-04 PET/CT scanning in the Nuclear Medicine Department of our hospital within 3 days from September 2020 to January 2021. We selected the data in which ovaries showed abnormal FDG activity. Patients with abnormal ovarian FDG uptake with focus confirmed by pathological biopsy or clinical follow-up as pathological changes were excluded. The uptake of tracers (^18^F-FDG and ^68^Ga-FAPI) was semi-quantitatively analyzed.

**Results:** This study included 14 patients (average age was 38.6). Physiological ovarian uptake was mostly unilateral, and there was no significant difference in SUVmax between the left and right sides (FDGt = 0.272, FAPIt = 0.592). The ovary SUVmax of FDG (4.89 ± 1.84) was statistically significantly higher than that of FAPI (1.53 ± 0.37). The Le/Li ratio on FDG is 3.38 ± 1.81, TBR is 5.81 ± 1.98, while the Le/Li ratio on FAPI is 3.57 ± 1.26, TBR is 0.94 ± 0.19.

**Conclusion:** Our research shows that ovarian functional or pathological changes can be manifested as FDG avid, while ^68^Ga-DOTA-FAPI-04 has no physiological accumulation in the ovary and is not affected by the menstrual cycle. Therefore, ^68^Ga-DOTA-FAPI-04 has unique advantages in the diagnosis of ovarian diseases, and can identify them early and accurately.

## Introduction

2-deoxy-2-[^18^F]fluoro-D-glucose (^18^F-FDG), known as “the century molecule,” is currently the most widely used oncological tracer. ^18^F-FDG positron emission tomography/computed tomography (PET/CT) is a valuable molecular imaging method widely used in the clinical diagnosis, staging and efficacy monitoring of various diseases. However, due to the pathological characteristics of glucose metabolism, ^18^F-FDG is highly distributed in normal organs including the brain, heart, and liver (1–3). Many physiological variations and pitfalls of whole-body ^18^F-FDG PET/CT imaging have been reported (4–6). In the pelvic region, activity retention within the urinary tract and ^18^F-FDG uptake in the normal intestine over very short segments are common sources of false-positives (7). At the same, ^18^F-FDG PET/CT lacks specificity in differentiating inflammation (8–10).

The menstrual cycle complicates the imaging of the female reproductive system. Gynecologic malignancies, including cervical, endometrial, vulvar, and ovarian carcinoma show ^18^F-FDG activity (11–13). ^18^F-FDG uptake in follicular ovarian cysts or hemorrhagic luteal cysts has previously been reported (13–15)]. In recent years, there have been reports of focal ^18^F-FDG uptake in the ovaries and uterus associated with the menstrual cycle in premenopausal women (14, 16–18), which may result in false positives on ^18^F-FDG PET imaging or malignancy being overlooked for physical uptake. This may be a challenge to nuclear medicine physicians. In order to identify if ovarian ^18^F-FDG uptake is physiological or pathological, the traditional method requires ^18^F-FDG PET/CT in different menstrual periods, which is not conducive to early clinical diagnosis. This has led to the development of more tracers.

Quinoline-based ligands targeting cancer-associated fibroblasts are promising radiopharmaceuticals in multiple tumors. Fibroblast activation protein (FAP) is a type II transmembrane glycoprotein expressed in dimer form on the surface of the tumor-associated cell matrix (CAFs) (19, 20). Evidence indicates that FAP is highly expressed in a variety of tumors, especially colorectal, ovarian, pancreatic, and hepatocellular carcinomas characterized by a strong desmoplastic reaction (21). CAFs with high FAP expression are associated with an adverse prognosis by promoting invasion, angiogenesis, micro-environmental immune suppression, and metastasis (22). ^68^Ga-DOTA-FAPI-04 showed the most favorable PET imaging properties, including low nanomolar affinity to FAP, near-complete internalization of FAP-bound radioactivity, and rapid blood clearance (23). This is a promising diagnostic and therapeutic target because of its low uptake in normal tissues and high target/non-target ratio.

To our knowledge, there have been no systematic investigations of ovarian FAPI metabolism. Herein, we present the results of a retrospective study of FAPI ovarian uptake patterns, which could help identify physiological and pathological changes for disease staging and formulate optimal treatment strategies.

## Materials and Methods

This is a retrospective analysis of a sub-cohort of patients from a previously acquired prospective database. Data were screened from the study previously registered at the clinical trial center and approved by the Clinical Research Ethics Committee of our Hospital. The study was conducted in accordance with the 1964 declaration of Helsinki and its subsequent amendments or similar ethical standards. In all cases, PET/CT scans were performed according to clinical needs or other protocols approved by our institutional review committee.

### Patients

We retrospectively analyzed the images of patients who simultaneously underwent ^18^F-FDG and ^68^Ga-DOTA-FAPI-04 PET/CT examination in the Department of Nuclear Medicine in our institution between September of 2020 and January of 2021. We selected the patients whose ovary demonstrated ^18^F-FDG uptake and reviewed their medical records and imaging findings. We then clarified any vague information to determine the reason for the increased FDG uptake. Pathological or imaging follow-up was the final determinant. The inclusion criteria were as follows: (1) female patients over 18 years old; (2) The inspection interval between FDG and FAPI is <3 days; (3) ovarian abnormal uptake on ^18^F-FDG; and (4) ovarian lesions were excluded by imaging and clinical follow-up or pathological results. The exclusion criteria were as follows: (1) history of ovarian tumors or related diseases and (2) patients without menstrual records or who failed to follow up. The first day of the menstrual cycle was recorded and all menstrual cycles were converted to a standardized 28 days for comparison.

### PET/CT Imaging

^18^F-FDG was manufactured per the standard method using the coincidence ^18^F-FDG synthesis module [FDG-N, PET (Beijing) Science and Technology, Beijing, China]. We purchased the precursor FAPI-04 from MedChemExpress LLC (Shanghai, China) with a purity of 98%. Radiolabeling of DOTA-FAPI-04 was performed by adding 1 mL sodium acetate (0.25 M) and 4 mL ^68^Ga-solution (370 MBq) to a reactor with a 25μg precursor FAPI-04. The final pH was ~4.0. The reaction was heated at 95°C for 10 min and the product was purified using a Sep-pak18C column. The final product was diluted with saline and sterilized by passing through a 0.22μm Millipore filter. The radiochemical purity was over 98% for ^18^F-FDG and ^68^Ga-DOTA-FAPI-04.

All patients were required to fast for at least 6 h before the ^18^F-FDG PET/CT examination. Serum glucose values were normal before the injection. No special preparation was required before ^68^Ga-DOTA-FAPI-04 PET/CT imaging (such as fasting or normal blood glucose levels). The dosage of intravenously injected ^18^F-FDG and ^68^Ga-DOTA-FAPI-04 was calculated based on the patient's weight (5.55 MBq [0.15mCi]/kg for FDG; 1.85 MBq [0.05mCi]/kg for FAPI).

Acquisition of ^18^F-FDG and ^68^Ga-DOTA-FAPI-04 imaging was started ~60 min after intravenous injection. The whole-body inspection scope was from the base of the skull to the base of the thigh. CT scan parameters included a tube voltage of 120 kV, a current of 120 mA, and a slice thickness of 3 mm. A PET scan in 3D acquisition mode was immediately performed after the CT scan and 5–6 beds were used depending on body length (90 s/bed for FDG and 3 min/bed for FAPI).

### Imaging Review

The Advantage Workstation was used to reviewed PET, CT, and fused PET/CT images. ^18^F-FDG and ^68^Ga-DOTA-FAPI-04 PET/CT scans were interpreted by two experienced board-certified nuclear medicine physicians. To prevent any bias, the research was reviewed in groups by study type: all ^18^F-FDG PET/CT images were reviewed by Tang W. and Wang Q. as group 1, and all ^68^Ga-DOTA-FAPI-04 PET/CT images were reviewed by Yang S. and Liu L. as group 2. Reviews were performed without other imaging data.

For a semi-quantitative analysis, regions of interest were manually drawn on transaxial images around the metabolic lesions of the uterine adnexa. The maximum standardized uptake value (SUVmax) was automatically calculated by the Advanced Workstation. The SUVmax of adjacent pelvic muscle was selected as the activity background, and the SUVmax of the ovary was divided by this muscle SUVmax to calculate target-to-background ratio (TBR). The mean standardized uptake value (SUVmean) of a round sphere with a diameter of 2 cm was selected from the liver to calculate Le/Li ratio (Le = ovary Li = Liver).

### Statistical Analysis

Statistical software package SPSS (IBM SPSS Statistics, Version 22) was used for data analysis and description. Descriptive statistics such as absolute and relative frequencies for discrete parameters and mean and standard deviation for continuous parameters were computed. Pearson correlation coefficient was used to describe the relationship between FDG-dose/FAPI-dose and total SUV. Results with a *P*-value <5% were statistically significant.

## Results

A total of 78 female patients underwent ^18^F-FDG and ^68^Ga-DOTA-FAPI-04 PET/CT examination within 3 days. Ovarian uptake was observed on ^18^F-FDG PET/CT images in 29 patients, 15 of whom were confirmed by pathology (*n* = 11) or follow-up (*n* = 4). This retrospective analysis included 14 patients with a mean age of 38.6 (range, 19–52 years). They were mainly in secretory phase (11/14 for FDG, 12/14 for FAPI) and proliferative phase (3/14 for FDG, 2/14 for FAPI). The demographic and clinical characteristics of the subjects are summarized in Table 1.

**Table 1:** The demographic and clinical characteristics of patients.

**Patient no**.	**Age (years)/Gender**	**Height (cm)**	**Weight (kg)**	**Primary diagnosis**	**Treatment**
1	19/female	152	38	Lymphoma	Radiotherapy and chemotherapy
2	27/female	155	56	Thyroid Ca	Surgery
3	29/female	162	55	Unknown fever	Conservative treatment
4	30/female	159	58	Trichoblastoma	Surgery
5	33/female	163	64	Lung Ca	Surgery
6	38/female	157	54	Thyroid Ca	Surgery
7	39/female	152	52	Stomach Ca	Surgery
8	42/female	162	61	Lung Ca	Surgery
9	45/female	158	60	Breast Ca	Surgery and chemotherapy
10	45/female	164	74	Lymphoma	Chemotherapy
11	46/female	156	72	Cervical Ca	Radiotherapy and chemotherapy
12	48/female	155	48	Breast Ca	Chemotherapy and Interventional therapy
13	48/female	165	78	Lung Ca	Surgery
14	52/female	158	69	Thyroid Ca	Surgery

**Figure 1:**
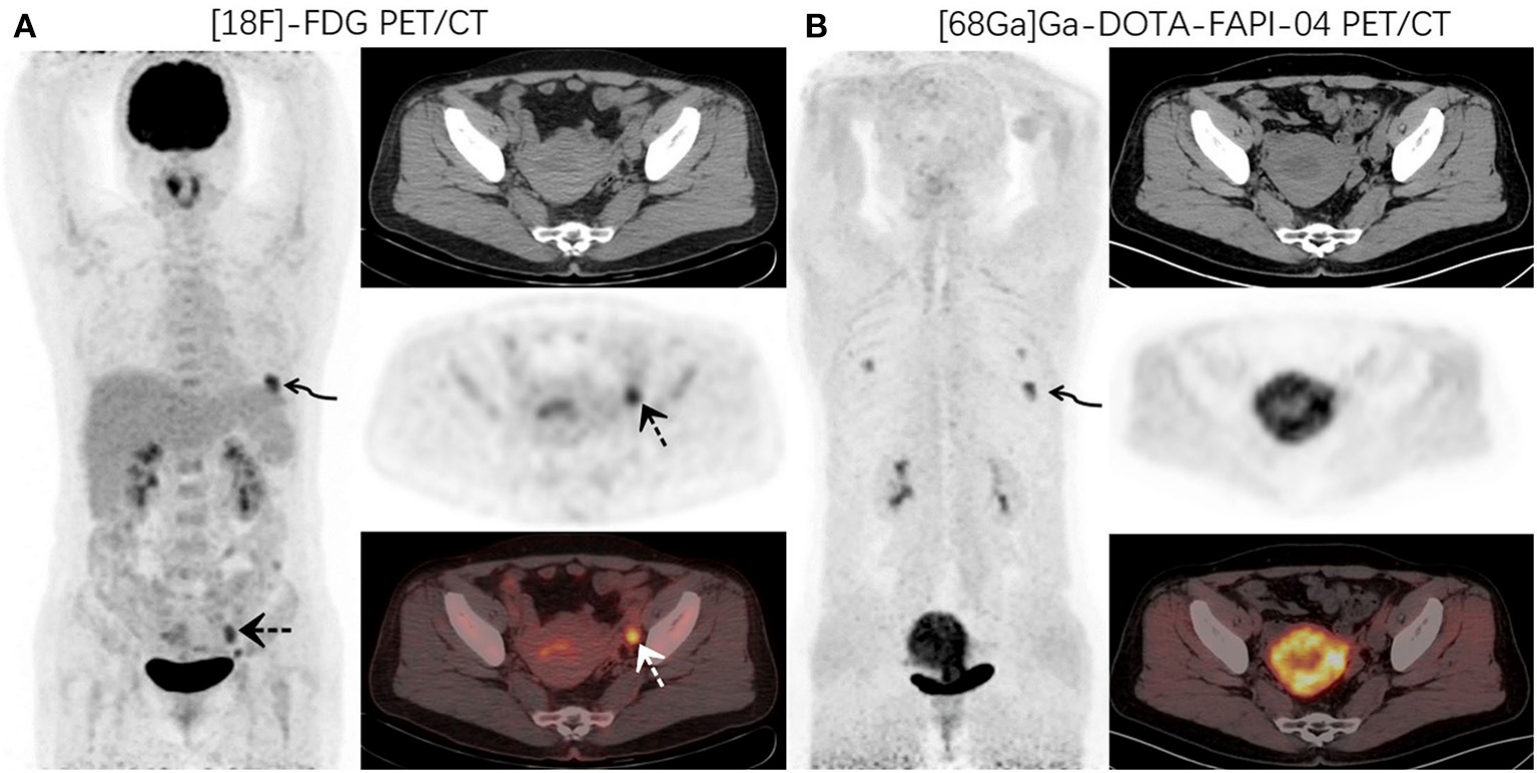
A 48-year-old woman with newly diagnosed lung cancer underwent a PET/CT scan for tumor staging. ^18^F-FDG PET/CT **(A)** images revealed increased FDG uptake in the left lung (curved arrow, SUVmax9.1). Abnormal activity was also observed in the endometrium and left adnexal area (dotted arrow, SUVmax 5.2). ^68^Ga-DOTA-FAPI-04 PET/CT **(B)** showed increased FAPI uptake in the left lung lesion (curved arrow, SUVmax10.6) and uterus. No other abnormal lesions were observed.

**Figure 2:**
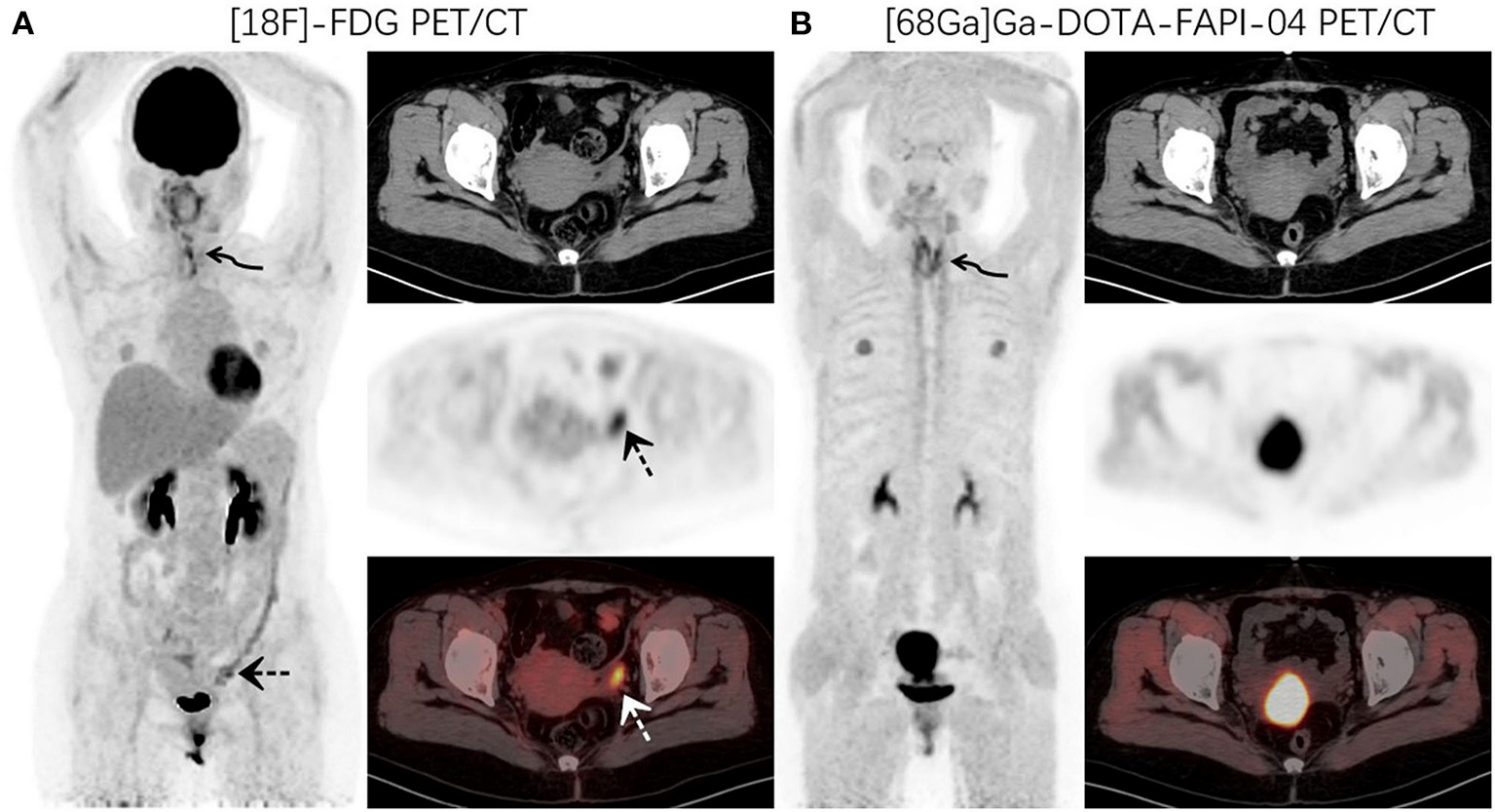
PET/CT was performed for restaging in a 52-year-old woman who underwent thyroidectomy for thyroid cancer 1 year prior. ^18^F-FDG PET/CT **(A)** images showed increased FDG uptake in the thyroidectomy area (curved arrow, SUVmax7.4) and left adnexal region (dotted arrow, SUVmax7.0). ^68^Ga-DOTA-FAPI-04 PET/CT **(B)** images demonstrated increased uptake of imaging agents in the thyroid area (curved arrow, SUVmax8.9), but no abnormal uptake was observed in the left adnexal region.

**Figure 3:**
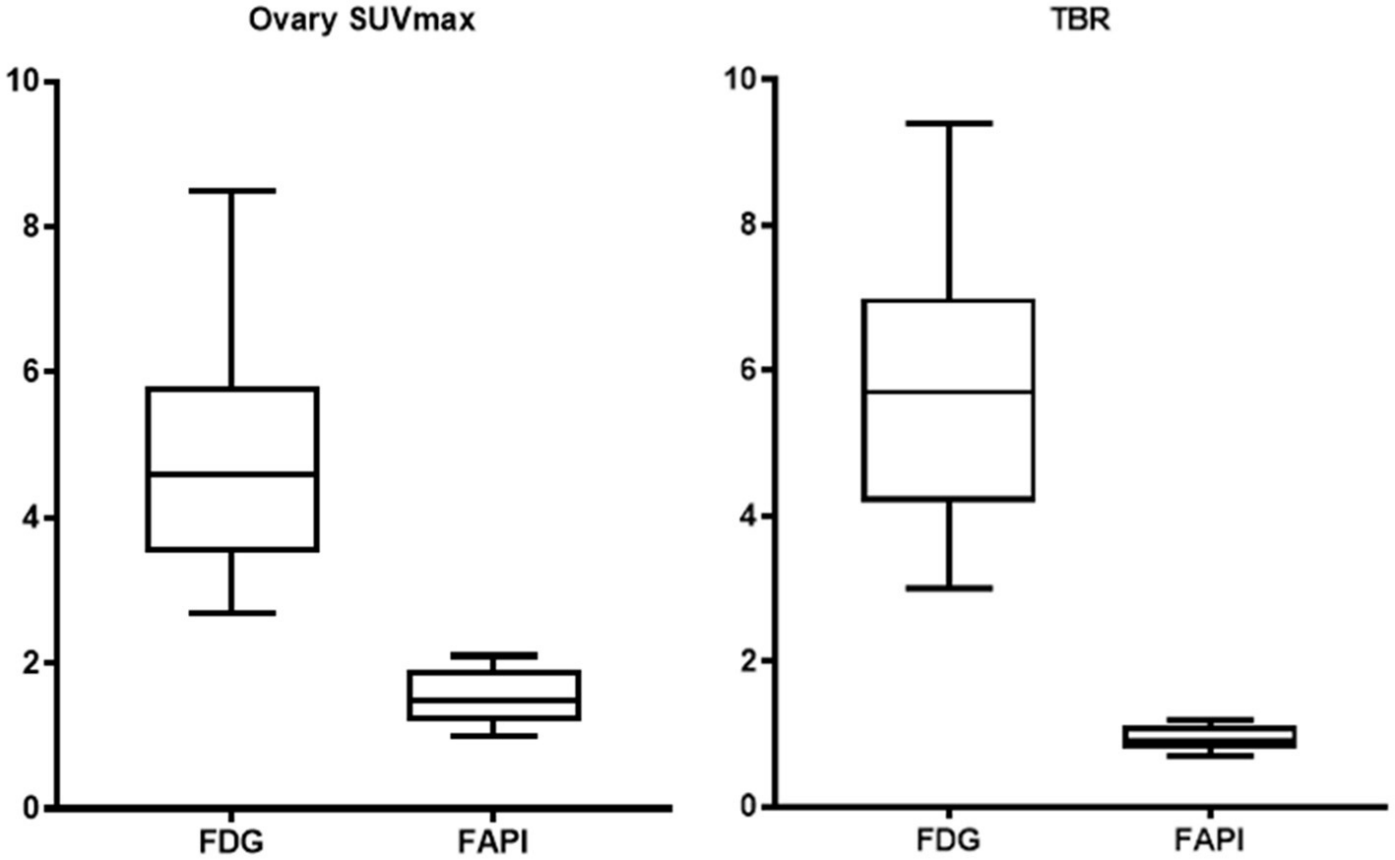
Comparison of FDG and FAPI uptake.

The FDG and FAPI PET/CT images showed that there were significant differences in the ovarian uptake between the two groups, most being unilateral. There was no significant difference in SUV between the left and right ovary (*t* = 0.272 for FDG, and *t* = 0.592 for FAPI). For the FDG group, the ovary SUVmax (4.89 ± 1.84) was statistically significantly higher than that of liver (1.57 ± 0.44) and pelvic muscle (0.86 ± 0.19) (*P* < 0.05). The average Le/Li ratio and TBR were 3.38 ± 1.81, and 5.81 ± 1.98, respectively. Physiological FDG uptake is associated with the menstrual cycle and occurs mainly in late hyperplasia and early secretion (Table 2). There was no significant difference between the two phases (*t* = 1.26, *p* = 0.23). Focal endometrial FDG uptake was observed in 4 patients (median SUVmax4.5, Figure 1). For the FAPI group, the average SUV of ovary, liver and muscle were 1.53 ± 0.37, 0.45 ± 0.10, and 1.64 ± 0.29, respectively. The difference between ovary and liver was statistically significant (*t* = 11.108, *p* < 0.05), while the difference between ovary and muscle was not (*t* = −1.33, *p* = 0.21). The average Le/Li ratio and TBR were 3.57 ± 1.26, and 0.94 ± 0.19, respectively. The uterus of the 14 subjects showed intense FAPI activity (average SUVmax 12.7, Figure 2). In addition, abnormal FAPI uptake in the broad ligament of the uterus was observed in a patient, which was manifested as a stripe of increased FAPI-avid (SUVmax 3.9). The TBR of the two groups was statistically different (*t* = 9.42, *p* < 0.05), while Le/Li ratio was not (Table 3). This is because normal liver has a high FDG uptake and a low FAPI uptake (one of the advantages of FAPI in displaying lesions, Figure 3).

**Table 2:** Summary of ^18^F-FDG and ^68^Ga-DOTA-FAPI-04 PET/CT images.

**Patient no**.	**Lesion size (mm^**2**^)**	**FDG**				**FAPI**			
		**Menstrual cycle (days)**	**SUVmax of ovary**	**Le/Li ratio**	**TBR**	**Menstrual cycle (days)**	**SUVmax of ovary**	**Le/Li ratio**	**TBR**
1	10.2 × 8.9	−14	4.1	4.6	8.2	−12	1.2	3.0	0.8
2	9.5 × 13.1	−17	3.9	3.3	5.6	−14	1.2	2.4	0.8
3	10.3 × 9.6	−12	4.6	3.5	6.6	−11	1.4	3.5	0.8
4	13.0 × 12.0	−11	8.5	8.5	9.4	−10	2.1	4.2	1.2
5	8.9 × 7.2	−13	3.6	2.6	6.0	−11	1.8	3.6	1.1
6	11.4 × 9.6	−8	3.3	2.5	4.1	−7	1.6	2.7	0.8
7	21.1 × 19.0	−11	8.2	5.5	9.1	−10	1.9	3.2	0.9
8	12.2 × 10.3	−12	4.6	2.4	4.6	−11	1.3	2.6	0.7
9	10.3 × 11.0	−16	4.6	2.2	4.2	−14	1.1	3.7	0.9
10	19.5 × 20.2	−15	2.7	1.5	3.0	−13	1.0	2.5	0.7
11	7.9 × 7.6	−10	5.4	2.6	5.4	−9	1.7	5.7	1.2
12	11.8 × 9.3	−14	2.8	1.8	3.5	−13	1.2	2.4	1.1
13	10.0 × 15.2	−11	5.2	3.5	5.8	−9	1.9	3.8	1
14	20.0 × 12.8	−13	7.0	2.9	5.8	−12	2.0	6.7	1.2

**Table 3:** Comparison of indicators between FDG and FAPI.

	**Ovary SUVmax**	**Liver SUVmax**	**Muscle SUVmax**	**Le/Li ratio**	**TBR**
FDG	4.89 ± 1.84	1.57 ± 0.44	0.86 ± 0.19	3.38 ± 1.81	5.81 ± 1.98
FAPI	1.53 ± 0.37	0.45 ± 0.10	1.64 ± 0.29	3.57 ± 1.26	0.94 ± 0.19
*t*	7.97	−8.30	8.39	−0.34	9.42
*P*-value	<0.05	<0.05	<0.05	0.74	<0.05

## Discussion

Both pathological and functional ovarian lesions can show abnormal FDG uptake. Physiologically, FDG accumulates in the female reproductive system, with a high SUV, making it difficult to obtain a clear image with high tumor-background contrast and increasing the diagnostic difficulty. In 2002, Chander et al. confirmed the physiological uptake of the endometrium and ovary in a serial PET/CT follow-up of a patient, and suggested that this performance varies with the menstrual cycle (17, 24, 25). Glucose phosphorylation is an important rate-limiting step in the estrogenic stimulation of uterine glycolysis (14). Hughes assessed endometrial enzymes activity in 252 patients with normal menstrual histories and found that, in normal endometrial tissue, glycogen synthetase activity synthesizes glycogen from glucose in increasing amounts until the midcycle (26). Glycogen phosphorylase then-breaks down glucose during the regressive stage of endometrial activity, causing decreased glycogen levels at the end of the cycle. Increased metabolic demands and inflammatory responses before and after ovulation may lead to increased ovarian FDG uptake. However, not all women with active menstruation experience increased FDG uptake for reasons that remain unclear. This physiological FDG uptake mainly occurs during the late follicular to early luteal phase of the menstrual cycle and is usually round or elliptical, mostly unilateral, and with a SUVmax >3 (25). Subsequent research has evaluated the characteristics of physiological FDG uptake in the ovaries, including other imaging methods and the differential diagnostic value of delayed imaging (17, 24, 25).

Cook et al. warn that a high uptake in the periphery of a benign cyst, such as a follicular ovarian cyst, may mimic a necrotic lymph node (15). Therefore, it is crucial to differentiate between physiological and pathological uptake. Measurement of the serum levels of menstrual cycles or ovarian hormones may help diagnose false positives but does not rule out pathological metabolism. The traditional method to distinguish the physiological and pathological uptake of the ovary requires repeated FDG examination in different physiological cycles, which may delay diagnosis.

SUVmax is the most commonly used index for evaluating metabolism on PET/CT, but differences in nuclides, patients, and equipment may cause certain differences. Therefore, using the target/non-target ratio is obviously more comparable. TBR can better perform semi-quantitative analysis of the uptake of different patients and imaging agents. Research has demonstrated that TBR has independent prognostic abilities for many lesions (27).

This retrospective analysis sought to evaluate the benefit and impact of ^68^Ga-FAPI-PET/CT in a small cohort of patients harboring suspicious ovarian lesions. We retrospectively analyzed the PET/CT images of 14 patients with suspected FDG false-positive ovaries. In contrast to ^18^F-FDG, ^68^Ga-DOTA-FAPI-04 has no physiological accumulation in the ovaries and is not affected by the physiological cycle, resulting in higher image contrast and better lesion delineation in the adnexal area of the uterus (Figure 3). ^68^Ga-DOTA-FAPI-04 positron emission tomography produces accurate and comprehensive imaging that can help determine the best treatment strategy. It may improves tumor staging, relapse monitoring, and necessary therapeutic interventions. Tumor lesions exceeding 1–2 mm in size require a supporting stroma (28). As the stroma volume can be larger than the tumor volume, stroma-targeted PET imaging may be more sensitive than glycolysis PET imaging for detecting small lesions with sufficient FAP-expressing stroma (22, 28). In 2019, Clemens et al. quantified the tumor-uptake in FAPI-PET/CT of various primary and metastatic tumors and found that ovarian cancer showed a moderate uptake of FAPI (SUVmax 6-12) (29).

### Limitations

This study has some limitations. First, the sample size was small and the patients varied greatly by primary disease (heterogeneity). Patient demographic characteristics may not reflect the general population. Second, this was a retrospective study. Some lesions might have been mistaken as physiological ingestion due to no obvious symptoms during follow-up. Currently, there is no literature evaluating FAPI's role in the ovaries. Therefore, prospective studies involving more patients are warranted to further explore ovarian FAPI uptake patterns.

## Conclusions

Both malignant and functional ovarian lesions can exhibit abnormal FDG uptake. ^68^Ga-DOTA-FAPI-04 has no physiological uptake and is not affected by the physiological cycle. It has a unique advantage in the diagnosis of ovarian diseases and can accurately differentiate physiological and pathological ovarian lesions in the early stage.

## Data Availability Statement

The original contributions presented in the study are included in the article/supplementary material, further inquiries can be directed to the corresponding author.

## Ethics Statement

Written informed consent was obtained from the individual(s) for the publication of any potentially identifiable images or data included in this article.

## Author Contributions

QW, SY, WT, LL, and YC: conception and design. QW: methodology, formal analysis, and writing–original draft. All the authors revised the paper, agreed to the submission of the final version of the manuscript, vouch for the accuracy and completeness of the data, analyses and for the fidelity of this report.

## Conflict of Interest

The authors declare that the research was conducted in the absence of any commercial or financial relationships that could be construed as a potential conflict of interest.

## Publisher's Note

All claims expressed in this article are solely those of the authors and do not necessarily represent those of their affiliated organizations, or those of the publisher, the editors and the reviewers. Any product that may be evaluated in this article, or claim that may be made by its manufacturer, is not guaranteed or endorsed by the publisher.
